# iGEAK: an interactive gene expression analysis kit for seamless workflow using the R/shiny platform

**DOI:** 10.1186/s12864-019-5548-x

**Published:** 2019-03-06

**Authors:** Kwangmin Choi, Nancy Ratner

**Affiliations:** Division of Experimental Hematology and Cancer Biology, Cincinnati Children’s Hospital Medical Center, Cincinnati, OH 45229 USA

**Keywords:** Transcriptomics, R, Shiny, Genomics, Graphical user interface, Microarray, RNA-seq, Differential expression (DE) analysis, Pipeline

## Abstract

**Background:**

The use of microarrays and RNA-seq technologies is ubiquitous for transcriptome analyses in modern biology. With proper analysis tools, the differential gene expression analysis process can be significantly accelerated. Many open-source programs provide cutting-edge techniques, but these often require programming skills and lack intuitive and interactive or graphical user interfaces. To avoid bottlenecks impeding seamless analysis processing, we have developed an Interactive Gene Expression Analysis Kit, we term iGEAK, focusing on usability and interactivity. iGEAK is designed to be a simple, intuitive, light-weight that contrasts with heavy-duty programs.

**Results:**

iGEAK is an R/Shiny-based client-side desktop application, providing an interactive gene expression data analysis pipeline for microarray and RNA-seq data. Gene expression data can be intuitively explored using a seamless analysis pipeline consisting of sample selection, differentially expressed gene prediction, protein-protein interaction, and gene set enrichment analyses. For each analysis step, users can easily alter parameters to mine more relevant biological information.

**Conclusion:**

iGEAK is the outcome of close collaboration with wet-bench biologists who are eager to easily explore, mine, and analyze new or public microarray and RNA-seq data. We designed iGEAK as a gene expression analysis pipeline tool to provide essential analysis steps and a user-friendly interactive graphical user interface. iGEAK enables users without programing knowledge to comfortably perform differential gene expression predictions and downstream analyses. iGEAK packages, manuals, tutorials, sample datasets are available at the iGEAK project homepage (https://sites.google.com/view/iGEAK).

## Background

The use of microarrays and RNA-seq technologies is ubiquitous in modern biology. A researcher uses at least one analysis pipeline or workflow to grasp high-impact information from these high-throughput data. However, conclusions are usually reached only after multiple iterations between analysis steps. For example, a researcher often needs to interactively evaluate, visualize, then assess the impact of selecting different parameters and cutoffs, and finally explore the overlapping consensus of cross-validated results obtained with different methods.

Proper analysis tools are necessary for accelerating the whole data analysis process. A researcher may choose open-source or commercial programs for mining data. Open-source programs are free and often provide cutting-edge data analysis techniques, but often require a certain level of programming skills. A lack of interactive graphical user interface is another issue. For example, R/Bioconductor project (https://www.bioconductor.org) provides invaluable open-source gene expression and downstream analysis tools/databases for both computational and experimental biologists but provides no or poor graphical user interfaces (GUI) to make analysis pipelines interactive and intuitive. This causes bottlenecks impeding seamless analyses in many biology labs. Commercial programs often provide well-designed user interfaces, but they are usually expensive and do not always guarantee better performance than open-source programs.

To address these challenges, we have developed an R/Shiny-based Interactive Gene Expression Analysis Kit (iGEAK), focusing on usability and interactivity. iGEAK is designed to be a simple, clean, light-weight open-source solution that contrasts with complicated and heavy analysis programs.

### Implementation

#### Comparisons with similar software programs

Integrative pipeline platforms for data analysis generally increase the software’s usability but often sacrifice efficiency and flexibility. For example, Galaxy [1] is a very flexible and popular bioinformatics platform and a reasonable choice for a specific single task (e.g. read count normalization), but its highly modular design requires different input files for separate analysis tasks. As a result, Galaxy is not ideal to design a seamless analysis pipeline since its users have to handle input/output file conversion, parameter settings, and filtering criteria for each step. In addition, installing Galaxy on a local machine is not feasible to most unskilled users.

Chipster [2] provides a versatile analysis platform with an intuitive graphical user interface and a rich collection of analysis tools for microarray and NGS data. However, users must download a pre-packaged virtual machine containing all the required components and set up a local server (Linux or Mac). The installation and configuration steps are not feasible for users unfamiliar with computer hardware and operating systems.

There are also R- (https://www.r-project.org) or Shiny- (https://shiny.rstudio.com) based server-side web applications dedicated to the transcriptome analysis. DEIVA [3], RNASeqGUI [4], The START App [5], and DEApp [6] provide interactive graphical interfaces for several R/Bioconductor packages. These and similar R/Shiny tools provide a limited workflow, and it is difficult to save analysis results. Furthermore, these web-based applications require users to upload data to a hosting server; uploading clinical data to these web applications might violate the Health Insurance Portability and Accountability Act of 1996 (HIPPA, https://www.hhs.gov/hipaa). In contrast, Pivot [7] is a desktop software with rich features, but users still need a basic knowledge of R and RStudio (https://www.rstudio.com) to install required libraries/packages and run the program. This is a substantial huddle for users unfamiliar with R and RStudio. Finally, all these programs handle only RNA-seq data, not Microarray data.

#### Design concept

iGEAK is the outcome of close collaboration with wet-bench biologists who are eager to easily explore, mine, and analyze unpublished or public microarray and RNA-seq data. iGEAK was originally designed to find a best solution for balancing between usability and flexibility, and as a portable gene expression analysis pipeline tool providing essential analysis steps and a user-friendly interactive GUI. This enables users without programing knowledge to comfortably perform differential gene expression prediction and downstream analyses. iGEAK uses simple tab-delimited text/csv files as input and output format, so users, if necessary, can easily save, open, and edit them using any text editor or spreadsheet program.

Downloadable program packages, user’s manual, and video tutorials are available at the iGEAK project site (https://sites.google.com/view/iGEAK) and users’ questions or suggestions can be easily shared via iGEAK User Group (https://groups.google.com/forum/#!forum/igeak). iGEAK is an open-source program available under The GNU General Public License v3.0 (GPLv3, https://www.gnu.org/licenses/gpl-3.0.en.html).

#### R/shiny desktop platform and hassle-free installation

Shiny is a powerful software-development platform that enables developers to implement flexible and expandable interactive (web) applications entirely in R and JavaScript languages via powerful modern browsers. Briefly, a developer creates a user interface and server written in R and JavaScript and Shiny compiles R code into the HTML, CSS, and JavaScript. Shiny tools are commonly used to make server-side web-services. Here we used Shiny to design a stand-alone desktop application, so that iGEAK can be used without a network connection.

iGEAK uses a portable R and a portable Chrome browser, so that users do not need to install R, integrated development environment (IDE) for R (e.g. RStudio), R/Bioconductor packages / databases, nor web browsers separately. All required packages and external datasets are pre-compiled in the portable R and data folders; thus, users can use iGEAK immediately after uncompressing an iGEAK package (zip) file compatible to their operating system. Once unzipped into a client computer’s internal or external storage, iGEAK is ready to use without further steps. Even a USB memory stick (> 4GB) can hold the application.

iGEAK is supported by the 64-bit operating systems (OS) including Linux, Windows, and macOS. Since iGEAK is a client-side desktop application, its speed and performance will vary depending on a client computer’s hardware specification. Our performance tests (up to 200 RNA-seq samples) show that iGEAK works well on conventional office desktop or laptop computers with 8GB RAM and Intel Core i5 CPU. iGEAK is built on R v3.3.2, compatible with any OS supporting v.3.3.2 or higher, including Ubuntu Linux 13–17, Windows 7–10, and macOS 10.10–13. Pre-installed package versions and iGEAK session information can be retrieved in iGEAK’s “Introduction” tab.

Unlike many other stand-alone desktop R/Shiny programs (e.g. Pivot [7]), launching iGEAK does not require any type of R consoles or IDEs (e.g. RStudio). Instead, users can directly launch iGEAK by double-clicking an executable launcher icon. iGEAKs is provided as two separate programs: iGEAK_microarray and iGEAK_RNA-seq. A user needs to choose a correct program corresponding to input data.

## Results

### Input files

**Fig. 1:**
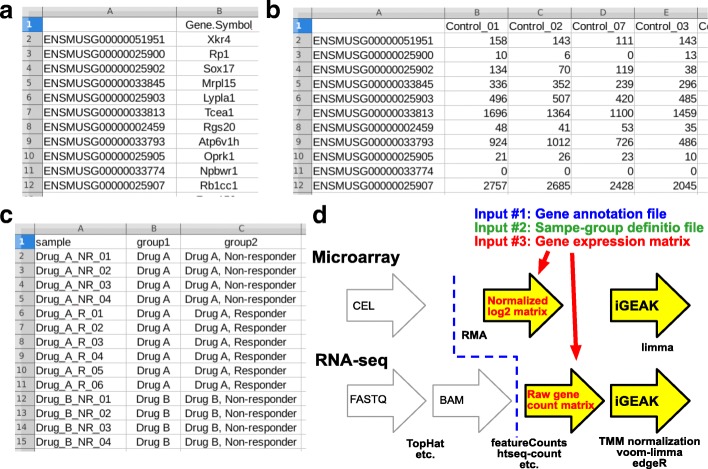
iGEAK requires 3 input files: a two-column gene annotation file (**a**), a gene expression matrix (**b**), and a multi-column sample-group metadata file (**c**). The data pre-processing steps to prepare input files are briefly explained as diagram (**d**). iGEAK does not directly process raw CEL files or FASTQ/BAM files (open arrows, **d**)

Three tab-delimited text/csv input files (gene annotation, sample-group definition, gene expression matrix) are required before running iGEAK (Fig. 1a-c), but file preparation is very straightforward (Fig. 1d). iGEAK does not directly use or process raw CEL (microarray) or FASTQ/BAM (RNA-seq) files. If a user has only raw files (CEL, FASTQ, BAM) and no bioinformatics skills, on-line tools such as ArrayAnalysis.org [8] and the Galaxy platform (https://usegalaxy.org) can be very useful to get a gene expression matrix. The Galaxy platform provides a variety of sequencing read aligners (e.g. TopHat (https://ccb.jhu.edu/software/tophat/index.shtml) or HISAT2 (https://ccb.jhu.edu/software/hisat2/index.shtml)) and read counting tools (e.g. featureCounts (http://bioinf.wehi.edu.au/featureCounts) or htseq-count (https://htseq.readthedocs.io/en/release_0.11.1)). iGEAK’s 3 input files can be directly prepared from this gene expression matrix and sample-group annotations (Fig. 1d).

**A "gene annotation" file** (Fig. 1a) is a tab-delimited two-column text/csv file. The first column is for unique gene identifiers, such as Affymetrix probeset IDs (microarray) or Ensembl gene IDs (RNA-seq). The second column should be always for gene symbols (or blank when no stable gene symbol is matched to a given unique gene ID). Various gene ID types can be converted into Gene symbols using DAVID (https://david.ncifcrf.gov/conversion.jsp) or BioDBnet (https://biodbnet-abcc.ncifcrf.gov/db/db2db.php). If raw gene counts (RNA-seq) were summarized by gene symbols, both columns should be gene symbols.

**A "sample-group definition" file** (or "metadata", Fig. 1c) is a tab-delimited multi-column text file. The first column describes sample IDs and all other columns contain sample group definitions. iGEAK can handle up to 10 different group definitions (columns). Users must choose one group definition column when this file is uploaded on to iGEAK.

**A "gene expression matrix" file** (Fig. 1b) is a tab-delimited data matrix of log2-transformed normalized gene expression values (microarray) or raw gene counts (RNA-seq). Detailed formats for the 3 input files are described at the iGEAK project site. Sample input files for microarray and RNA-seq studies are also available at the site.

### Tabs

**Fig. 2:**
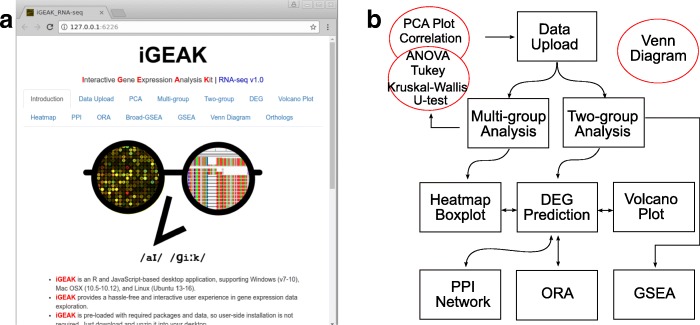
iGEAK’s analysis steps represented by “tab”s (**a**) and actual workflow (**b**). After locally uploading 3 input files, users can choose “Multi-group” analysis path including sample PCA analysis, sample correlation analysis, parametric (Analysis of Variance (ANOVA) / Tukey) tests, and non-parametric (Kruskal-Wallis / U-) tests. Users can also create heatmap and boxplots displaying gene expression levels across multiple sample groups. If users choose “Two-group” path, differentially expressed genes are first predicted (“DEG”) and this step subsequently affects “Heatmap”, “Volcano Plot”, “PPI”, and “ORA” tabs. iGEAK uses all genes from two sample groups to perform “GSEA” analysis and to generate 3 input files for the Broad-GSEA program

Each analysis step (Fig. 2a and b) corresponds to a “tab” in iGEAK’s GUI. When a user changes input data or parameters and clicks a tab, the tab (function) instantly updates outputs, so that a user can easily and quickly explore data using different combinations of parameters. Some tabs (“Introduction”, “Venn Diagram”, and “Orthologs”) are independent, but others inter-connected. Currently 14 tabs are implemeted in iGEAK. A detailed step-by-step tutorial (slides and video clip) using a sample RNA-seq dataset is available at the iGEAK project homepage (check the “Crash Course” section).

**“Introduction” tab:** This tab provides brief descriptions about iGEAK, the copyright disclaimer, and action buttons displaying pre-installed R/Bioconductor packages and iGEAK session information.

**“Data Upload” tab:** Three input files are uploaded to iGEAK using this interface. Users also need to choose species (human or mouse), sample groups, and parameters for filtering sub-optimal probesets (microarray) or low-count genes (RNA-seq). A normalized gene count matrix (RNA-seq) using the trimmed mean of M-values normalization (TMM) method [9] is also generated at this step.

**Fig. 3:**
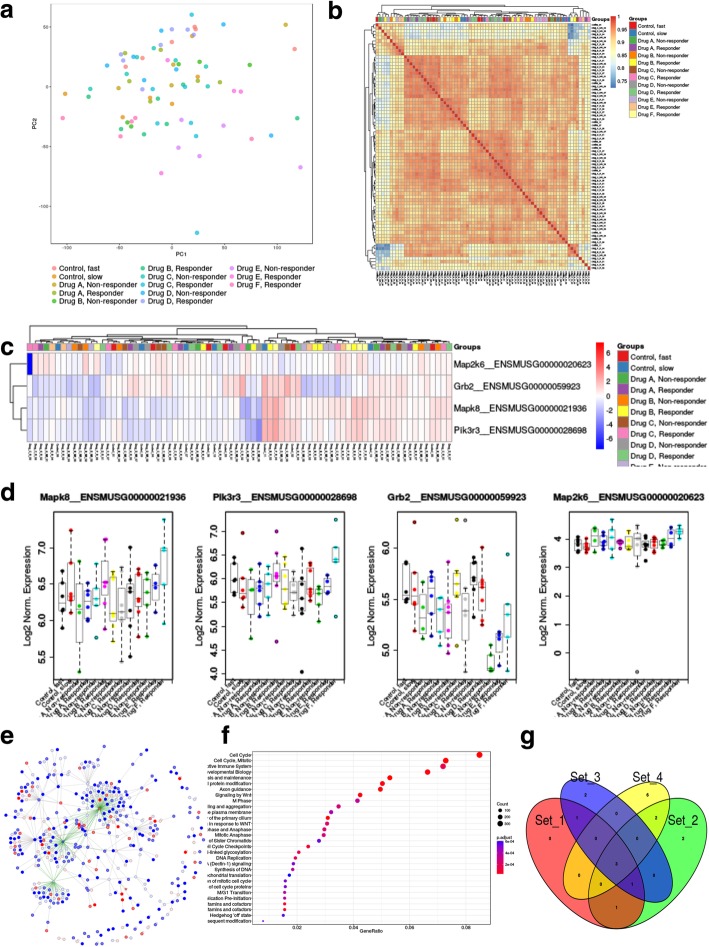
Examples of iGEAK’s customizable plot types: a principal component analysis (PCA) plot (**a**), a sample-correlation plot (**b**), a gene expression heatmap (**c**) and boxplots (**d**), a protein-protein interaction (PPI) graph (**e**), an over-representation analysis (ORA) report plot (**f**), and a multi-way venn diagram (**g**)

**“PCA” tab:** iGEAK creates an interactive principal component analysis (PCA) plot and a sample (Pearson) correlation plot based on transcriptomes (Fig. 3a and b). These two plots help users quickly and visually to identify outlier samples. When outlier samples are detected, users can easily re-group them by editing a group-definition file in a spreadsheet program (e.g. Excel) or a text editor, then re-upload the updated file to iGEAK.

“**Multi-group” tab:** This tool is designed for users who want to quickly check gene expression patterns (heatmap (Fig. 3c), boxplot (Fig. 3d), test statistics) of a given gene set across multiple samples and groups. Users can perform parametric tests (analysis of variance (ANOVA) and post-hoc Tukey’s test) or non-parametric tests (Kruskal-Wallis and Mann-Whitney U-test) based on the samples’ characteristics and research design/goals. To help users to choose between parametric and non-parametric test, iGEAK provides (1) Shapiro-Wilk Normality test statistics and (2) Group dispersion (= standard deviation for each group). If the Shapiro-Wilk test *p*-value > 0.05, the parametric tests (ANOVA and Tukey’s test) are preferred, as the expression data do not seem to violate the normality assumption. However, if each group’s sample size is > 15 and there are 2–9 groups in total, the parametric tests can perform well even with continuous data that are slightly non-normal. Users are recommended choosing the non-parametric tests (Kruskall-Wallis and post-hoc pairwise Mann-Whitney U-test) if the expression data violate the normality assumption and/or the total sample size is very small, but the data for all groups have the same dispersion. If sample groups have different dispersions, the non-parametric tests might not provide valid results.

**“Two-group” tab:** The most common microarray or RNA-seq data analysis is a two-group comparison. Users choose two sample groups here and this decision affects the following 5 tabs (“DEG”, “Heatmap”, “Volcano Plot”, “PPI”, and “ORA”) that use two sample groups. The two sample groups can be any subset of selected multi-groups from the “Data Upload” tab. During RNA-seq data analysis, raw gene counts from samples in two groups are re-processed for read count normalization.

**“DEG” tab:** To predict gene-level differentially expressed genes (DEGs), iGEAK uses the R/Bioconductor limma [10] package for microarray data and edgeR [9] or voom-limma [11] packages for RNA-seq data. For RNA-seq data analysis, users need to choose one method between edgeR and voom-limma. Once users set 3 filtering parameters (minimum fold, *p*-value, multiple testing), this tab reports a filtered DEG list and statistics, such as log2fold (logFC), p-value, adjusted *p*-values after a chosen multiple testing method. If users do not choose a method of multiple testing (“none”), adjusted p-values and original p-values are the same. The report table provides the URL links to NCBI Gene database (https://www.ncbi.nlm.nih.gov/gene) in the last column.

Three parameters used in the “DEG” tab affect “DEG”, “Volcano Plot”, “Heatmap”, “PPI”, and “ORA” tabs. To change output results from these tabs, users need to revisit the “DEG” tab to adjust filtering parameters. The “Broad-GSEA” and “GSEA” tabs are not affected by these parameters because the GSEA method uses all genes in the original expression data matrix.

**“Volcano Plot” tabs:** Differentially expressed genes are conveniently visualized using this interactive volcano plot. This tool is useful to visualize the distribution of DEGs in terms of log10 (adjusted) p-value and log2 fold change. Users can quickly identify extremely changed genes and get their differential expression information by setting a window area around genes using a mouse. This plot can be downloaded as an image file.

**“Heatmap” tab:** iGEAK provides a highly reconfigurable heatmap and boxplot generation tool. Users can easily adjust width, height, font size, tree height, scaling method, clustering type, and color using this tool. A heatmap is generated by clicking “Create/Reset” button. If no gene (symbol) of interest is submitted, a heatmap including all DEGs is generated. No boxplot is created because the total numbers of DEGs could be too many. If a subset of DEGs are submitted, a heatmap and boxplots are created. These plots can be downloaded as image files.

**“PPI” tab:** This tab is a protein-protein interaction (PPI) and transcription-control network visualization tool using the visNetwork (https://github.com/datastorm-open/visNetwork) package (Fig. 3e). The network nodes (proteins) and edge (interactions) are color-coded based on their fold change levels and interactions type (PPI, TF, PPI + TF), where TF represents “transcription factor (TF)– target interaction”.

Users can easily change network layouts, search genes, edit the network directly, and download it as an image file. This PPI network could be scarce if there are only small numbers of predicted DEGs. In this case, users may revisit the “DEG” tab and adjust the filtering parameters (i.e. lower minimum fold, higher *p*-value cutoff, no multiple testing) to get more DEGs.

The physical PPI information was extracted from BioGrid (https://thebiogrid.org, v3.4). Transcription factors (TFs) and target genes with conserved (human, mouse, rat, and dog) TF binding sites are also visualized. The backbone PPI network is extended by adding TFs (“star” shaped node) and genes having transcription factor binding sites (TFBS) within promoters and/or 3′-UTRs. This information is extracted from MSigDB’s C3 dataset (http://software.broadinstitute.org/gsea/msigdb). Currently, iGEAK provides human and mouse data only.

**“ORA” tab:** iGEAK provides over-representation analysis (ORA) based on the Reactome database (http://www.reactome.org) and the ReactomePA package [12]. A summary dot plot (top-30 highly enriched gene sets, Fig. 3f) and detailed enrichment result table are reported in this tab. This analysis uses DEGs predicted in the “DEG” tab. If no result is reported, users may need to adjust filtering parameters in the “DEG” tab to increase the numbers of DEGs.

**“GSEA” tab:** iGEAK provides a light-version of Gene Set Enrichment Analysis (GSEA) [13]. The current version is based on the Reactome database and ReactomePA package. Currently, iGEAK supports human and mouse data only. This tab is not affected by parameter settings in the “DEG” tab.

**“Broad-GSEA” tab:** For users who prefer a stand-alone Java-based GSEA program from Broad Institute (http://software.broadinstitute.org/gsea), iGEAK provides three GSEA input files (expression (txt), phenotype (cls), annotation (chip)). Users can choose many different reference gene set databases and refined functions / metrics in the Broad’s GSEA program. This tab is not affected by the “DEG” tab because all genes (not DEGs) are included in the files.

**“Venn Diagram” tab:** This venn diagram tool creates a highly reconfigurable 1- to 5-way Venn diagram (Fig. 3g) and a summary table showing genes in each section. Users can freely change group title, font size, plot size, color, and transparency and download a final Venn diagram as an image file. We re-wrote several functions in R’s original vennDiagram package for better plotting and data extraction.

**“Orthologs” tab:** Gene symbols are intuitive, but not ideal unique identifiers because they can change. The HUGO Gene Nomenclature Committee (HGNC, https://www.genenames.org) and The Mouse Genome Informatics Database (MGI, http://www.informatics.jax.org) are the authoritative resources of the official human and mouse genes and their updated gene symbols.

Converting gene symbols between human and mouse are sometimes tricky. When a human gene symbol and its mouse counterpart use the same letters, the only difference is that human gene symbols are all uppercase, but mouse gene symbols are lowercase except for the first letter. The first tool in this tab provides this simple function and is also useful to convert gene symbols into protein symbols (all uppercase, for human and mouse). However, in many cases, a human gene and its orthologous/homologous mouse genes have different gene symbols. iGEAK provides a parsed Ensembl-v92 dataset (https://useast.ensembl.org) to retrieve inter-species (i.e. between human and mouse) orthologs/homologs.

## Conclusion

iGEAK is a ready-to-use desktop software and hassle-free in installation and deployment. iGEAK exploits the power of R/Bioconductor packages and gives users full control over key analysis and plotting parameters. iGEAK’s intuitive and interactive design allows users to utilize it as an efficient data presentation and exploration tool for group discussion. iGEAK uses simple and small-sized text files as input/output format that can be easily shared between researchers. It is designed to be easily extendable and future versions are expected to integrate popular transcriptome analysis routines as they are made available to the research community.

## Abbreviations

ANOVA: ANalysis Of VAriance
DEG: Differentially Expressed Gene
GPLv3: GNU General Public License v3.0
GSEA: Gene Set Enrichment Analysis
GUI: Graphical User Interface
HGNC: HUGO Gene Nomenclature Committee
HIPPA: Health Insurance Portability and Accountability Act
IDE: Integrated Development Environment
iGEAK: Interactive Gene Expression Analysis Kit
MGI: Mouse Genome Informatics
ORA: Over-Representation Analysis
PCA: Principal Component Analysis
PPI: Protein-Protein Interaction
TF: Transcription Factor
TFBS: Transcription Factor Binding Site
TMM: Trimmed Mean of M-values
